# Invasive *Wickerhamomyces anomalus* Infections among Injecting Drug Users, France, 2012–2024^1^

**DOI:** 10.3201/eid3209.251817

**Published:** 2026-09

**Authors:** Maxime Lefranc, Adrien Pain, Fréderic Dalle, Pierre Baudino, Claudio Plaisant, Taieb Chouaki, Josephine Dorin, Anne-Pauline Bellanger, Julie Bonhomme, Maxime Moniot, Celia Rouges, Françoise Botterel, Gregoire Pasquier, Lilia Hasseine, Estelle Perraud-Cateau, Jean-Pierre Gangneux, Laurence Delhaes, Damien Costa, Juliette Guitard, Alexandre Alanio, Valerie Letscher-Bru, Emilie Guemas, Guillaume Desoubeaux, Karine Boukris-Sitbon, Olivier Lortholary, Fanny Lanternier, WAIT-IV Study Group,, Marie Desnos-Ollivier, Sebastien Imbert

**Affiliations:** CHU Bordeaux, Bordeaux, France (M. Lefranc, P. Baudino, C. Plaisant, L. Delhaes, S. Imbert); Université de Bordeaux, Bordeaux (M. Lefranc, S. Imbert); Institut Pasteur, Paris, France (A. Pain, K. Boukris-Sitbon, O. Lortholary, M. Desnos-Ollivier, F. Lanternier); CHU Dijon, Dijon, France (F. Dalle); CHU Amiens-Picardie, Amiens, France (T. Chouaki); CHU d’Antibes Juan les Pins, Antibes, France (J. Dorin); CHU de Besançon, Besançon, France (A.-P. Bellanger); CHU Caen, Caen, France (J. Bonhomme); CHU Clermont-Ferrand, Clermont-Ferrand, France (M. Moniot); AP-HP Hôpital Cochin, Paris (C. Rouges); CHU Henri Mondor, Créteil, France (F. Botterel); CHU de Montpellier, Montpellier, France (G. Pasquier); CHU Nice, Nice, France (L. Hasseine); CHU Poitiers, Poitiers, France (E. Perraud-Cateau); CHU de Rennes, Rennes, France (J.-P. Gangneux); CHU de Rouen, Rouen, France (D. Costa); Sorbonne Universite, Paris (J. Guitard); Hôpital Saint-Antoine, Paris (J. Guitard); Hôpital Saint-Louis, Paris (A. Alanio); Hôpitaux Universitaires de Strasbourg, Strasbourg, France (V. Letscher-Bru); CHU Toulouse, Toulouse, France (E. Guemas); CHU de Tours, Tours, France (G. Desoubeaux)

**Keywords:** Wickerhamomyces anomalus, fungi, invasive fungal infections, fungemia, drug users, whole-genome sequencing, France

## Abstract

*Wickerhamomyces anomalus* is a yeast rarely involved in human invasive fungal diseases (IFD). We retrospectively analyzed 44 episodes of W. anomalus IFD in France during 2012–2024. Injecting drug use (IDU) was the main risk factor among 26/35 (74.3%) incident cases. Most infections were community acquired; overall 3-month mortality rate was 1/30 (3.3%). Short tandem repeat (STR) genotyping and whole-genome sequencing analyses revealed substantial genetic diversity among isolates. However, 1 STR genotype was shared by 2 IDU patients, suggesting common exposure. In addition, 1 isolate obtained from a cotton filter used for drug preparation was identical by STR genotyping to the bloodstream isolate from the same patient, indicating direct inoculation via contaminated material or poor injection practices. Our findings highlight the increased risk for W. anomalus IFD among IDU patients and emphasize the importance of targeted preventive measures within that population.

*Wickerhamomyces anomalus* (syn. *Candida pelliculosa*, *Pichia anomala*) is a diploid, ubiquitous yeast species found in diverse environments including soil, fruits, trees, wastewater, marine ecosystems, and the digestive tracts of insects (*1*). It exhibits antimicrobial activity against other yeasts, filamentous fungi, and bacteria and possesses the ability to produce aromatic compounds and exoenzymes (*2*). Those properties make *W. anomalus* of notable interest for various biotechnological applications, including those in the food, environmental, industrial, and medical sectors, as well as in oenological biotechnology (*3*). 

*W. anomalus* is rarely isolated in clinical settings and is responsible for <1% of invasive fungal diseases (IFD) caused by yeasts (*4*–*6*). Nevertheless, hospital outbreaks of *W. anomalus* IFD have been documented, particularly in neonatal and pediatric intensive care units (ICUs) (*7*–*12*). In several instances, clonal transmission has been demonstrated, suggesting an exogenous source of contamination (*8*,*13*). Additional outbreaks have also been reported among immunocompromised adult patients (*13*–*17*). Furthermore, a recent nationwide retrospective cross-sectional study on fungemia among injecting drug users (IDUs) in France identified *W. anomalus* as overrepresented in IDU-related cases (*18*), reinforcing the hypothesis of exogenous contamination. However, in both hospital outbreaks and IDU cases, the precise source of infection remains unidentified.

In this study, we aimed to investigate factors associated with *W. anomalus* IFD in France. We conducted a comprehensive analysis of epidemiologic, clinical, and genetic characteristics of all *W. anomalus* IFD cases reported over a 13-year period nationwide, with particular emphasis on IDU-associated infections.

## Materials and Methods

### Study Design and Network 

**We analyzed IFDs caused by *W. anomalus* yeast diagnosed during January 2012–December 2024 in France and declared during the 2 consecutive prospective national surveillance programs RESSIF (RESeau de Surveillance des Infections Fongiques invasives**; **ended December 31, 2022) and SINFONI (**Surveillance des Infections FONgiques Invasives; **started January 1, 2023). The RESSIF multicentric surveillance program was described previously** (*4*)**.** SINFONI is a multicentric prospective program for surveillance of IFD started January 1, 2023, and active as of August 2026 involving 59 hospitals in France and overseas (Appendix Figure 1). Participants report all cases of IFD diagnosed in the hospital using an online secured REDCap questionnaire (https://project-redcap.org) designed and monitored at the National Reference Center for Invasive Mycoses and Antifungals (NRCMA; Institut Pasteur, Paris, France). **For both RESSIF and SINFONI surveillance programs, participants completed the** questionnaire **for each episode of IFD, reporting demographic data (age, sex, hospital), clinical information (risk factor for IFD, underlying conditions, preexposure to antifungals, first treatment for IFD, outcome) and microbiologic findings (species identification).**

The Institut Pasteur Institutional Review Board approved NRCMA surveillance activities (no. 2009-34/IRB), as did the Commission Nationale de l’Informatique et des Libertés (déclaration no. 351952). We designed and reported our study in accordance with Strengthening the Reporting of Observational Studies in Epidemiology (STROBE) guidelines (*19*).

### *W. anomalus* IFD Case Definitions

***W. anomalus* IFD cases included both fungemia and deep-seated infections. We defined fungemia by the isolation of *W. anomalus* yeast from a blood culture specimen. We defined deep-seated infections by the isolation of *W. anomalus* from a normally sterile site, regardless of whether fungemia was also present. We defined community-onset cases as episodes in which a positive blood culture for *W. anomalus* was obtained <3 days after hospital admission. We defined recurrent episodes as the reisolation of the same fungal species >10 days after the initial episode** (*18*)**.**

### Strain Identification

**We checked all *W. anomalus* isolates sent to the NRCMA on BBL CHROMagar *Candida* medium (**https://www.chromagar.com**) for purity, then identified them at the species level using a polyphasic approach combining matrix-assisted laser desorption/ionization time-of-flight (MALDI-ToF) mass spectrometry (Bruker Biotyper or Sirius devices; Bruker MBT Library,** https://www.bruker.com**), sequencing D1D2 and internal transcribed spacer (ITS) regions of the DNA ribosomal loci using panfungal primers NL1/NL4 and V9D/LS266** (*20*)**.** **We performed DNA extraction using NucleoMag Plant DNA extraction kit (Macherey Nagel,** https://www.mn-net.com**) in a Thermo KingFisher extractor (Thermo Fisher Scientific,** https://www.thermofisher.com**) preceded by 2 bead beating steps using ATL Buffer lysis (QIAGEN,** https://www.qiagen.com**) in lysing matrix tubes Y (MP Biomedicals,** https://flowsciences.com**) and a heating step in the presence of RNase A. Sequences of D1D2 and internal transcribed spacer regions of the isolates had a percentage identity >99% with the sequences of the type strain of *W. anomalus* CBS 5759, enabling species identification. We performed antifungal susceptibility testing in accordance with EUCAST broth microdilution reference method** (*21*)**.**

### Short Tandem Repeat Genotyping

We performed short tandem repeat (STR) genotyping as **previously described** (*22*)**,** **with slight modifications. In brief, we used a panel of 6 STR markers, 3 trinucleotide and 3 hexanucleotide repeats, resulting in a unique 12-marker profile for each isolate.** We amplified the 3 trinucleotide STRs in 1 multiplex PCR reaction and the 3 hexanucleotide STRs in 3 distinct simplex PCR reactions. We used GoTaq DNA polymerase (Promega, https://www.promega.com) for all amplifications. We then diluted PCR products 1:100 in water; we mixed 1 µL of the diluted products with 10.2 μL of formamide and 0.3 μL of MapMarker 1000 DNA size standard (Eurogentec, https://www.eurogentec.com). We incubated the mixtures at 95°C for 3 minutes and subsequently analyzed them using a 3500 XL Dx genetic analyzer (Thermo Fisher). We determined repeat numbers for each STR locus using GeneMapper 5 (Thermo Fisher). We converted the resulting STR profiles into a genetic distance matrix using the bruvo.dist function from the poppr package in R version 4.5 (The R Project for Statistical Computing, https://www.r-project.org) as previously described (*23*). We constructed an UPGMA tree via the bruvo.boot function to visualize genetic relationships among samples; we exported the resulting tree in Newick format and visualized it using the Interactive Tree of Life web interface (*24*).

### Whole-Genome Sequencing Analysis 

We sequenced whole genomes at the Mutualized Platform for Microbiology (P2M), Institut Pasteur, using an Illumina NextSeq 500 sequencer. We constructed libraries using Nextera DNA Library preparation kit (Illumina) and sequenced them using a 2 × 150 nt paired-end strategy. We also incorporated 11 publicly available isolates reprocessed with the same pipeline (*22*)**.** We assessed raw read quality using FastQC version 0.12.1 (http://www.bioinformatics.babraham.ac.uk/projects/fastqc) and MultiQC version 1.12 (https://github.com/MultiQC/MultiQC) (*25*). We performed read cleaning with AlienTrimmer version 2.0 (ftp://ftp.pasteur.fr/pub/gensoft/projects/AlienTrimmer) (*26*): we considered bases with a Phred score <15 (–q 15) of low confidence, and after trimming, we discarded reads <50 bases (–l 50) or containing >50% low-confidence bases (–p 50). Cleaned reads are available at the European Nucleotide Archive (accession no. PRJEB93917).

### Variant Calling and Population Genomic Analyses

We defined methods for variant calling and population genomic analyses (Appendix). In brief, we aligned reads to the *W. anomalus* KG16 reference genome assembly (GenBank no. GCA_019321675.1) and imported variant calls into R version 4.3.2 using the VCFR package as described previously (*27*). We converted the filtered VCF file to a genlight object (vcfR2genlight) and set diploidy. We computed pairwise genetic distances between isolates as the proportion of differing single-nucleotide polymorphisms (SNPs) using bitwise distances (bitwise.dist function in poppr package) (*23*), normalized by the reference genome size (13,671,194 bp; KG16 assembly). We constructed a neighbor-joining phylogenetic tree from the distance matrix using the nj function from the APE package as described (*28*). We assessed node support by bootstrap resampling 1,000 replicates using the aboot function in poppr package. We exported the final tree in Newick format and visualized it using Interactive Tree of Life web interface (*24*).

We assessed genetic diversity by computing Simpson diversity index (λ), Nei unbiased gene diversity (Hexp), and global genetic differentiation between phylogenetic clades (FST). We inferred population structure using ADMIXTURE (https://dalexander.github.io/admixture/index.html).

### De Novo Genome Assemblies and Loss of Heterozygosity Map

We performed de novo genome assemblies using the fq2dna pipeline (https://gitlab.pasteur.fr/GIPhy/fq2dna), which performs read deduplication, quality trimming, error correction, digital normalization, and de novo assembly with SPAdes (*29*). We assessed assembly quality using QUAST (*30*). We described methods to identify and visualize regions of Loss of heterozygosity (LOH) (Appendix); in brief, we processed allele depth matrices in R using dplyr (https://dplyr.tidyverse.org), tidyr (https://tidyr.tidyverse.org), and ggplot2 (*31*) as described previously. We stratified analyses and visualizations both globally and by predefined phylogenetic clusters.

### Statistical Analysis 

We summarized continuous variables as medians with interquartile ranges (IQRs), where appropriate, and categorical variables as counts and percentages. To avoid autocorrelation, we included only incident cases in the description of baseline characteristics. We compared continuous variables using the Wilcoxon rank-sum test and categorical variables using Fisher exact test. All statistical tests were 2-sided, and significance threshold was p<0.05. We conducted analysis using the gtsummary package in R version 4.5.

## Results

### *W. anomalus* IFD Episodes

During the study period, a total of 47 *W. anomalus* IFD episodes were reported to the NRCMA, 31 during the RESSIF program and 16 during the SINFONI program. We excluded 3 episodes from the study, 2 superficial ocular infections and 1 with inconsistent data, for a total of 44 episodes (40 fungemia and 4 osteoarticular infections) among 35 patients (Table). Episodes originated from 18 centers located across mainland France; each center reported 1–12 episodes. Among centers that participated exhaustively in both RESSIF and SINFONI surveillance programs (n = 15), we have observed an upward trend in the annual incidence of new cases since 2021; during 2012–2020 the mean was 2.2 reported cases per year, and during 2021–2024 the mean was 5.25 reported cases per year (Appendix Figure 2).

**Table:** Demographic and clinical characteristics of invasive *Wickerhamomyces anomalus* infection cases reported through multicentric prospective national surveillance programs, France, 2012–2024*

Characteristic	All patients, N = 35	IDU, n = 26	Non-IDU, n = 9	p value†
Age median (IQR)	42 (36–50)	41 (36–45)	55 (36–68)	0.11
Sex				
M	27/35 (77)	20/26 (77)	7/9 (78)	>0.9
F				
Invasive fungal disease				0.6
Fungemia	31/35 (89)	22/26 (85)	9/9 (100)	
Osteoarticular infection	4/35 (11)	4/26 (15)	0/9 (0)	
IFD risk factor other than IDU‡				
HIV infection	2/31 (6.5)	2/26 (7.7)	0/5 (0)	>0.9
Recent surgery	2/35 (5.7)	0/26 (0)	2/9 (22)	0.061
Central vascular catheter	9/35 (26)	2/26 (7.7)	7/9 (78)	<0.001
Intensive care unit	7/35 (20)	3/26 (12)	4/9 (44)	0.055
Other underlying conditions‡	4/35 (11)	1/26 (4)	4/9 (33)	0.010
>1 risk factor other than IDU	13/35 (37)	6/26 (23)	8/9 (89)	<0.001
IFD history	8/33 (24)	7/25 (28)	1/8 (13)	0.6
Community onset	17/34 (50)	16/26 (62)	1/8 (13)	0.039
Associated deep-seated IFD				0.8
Spondylodiscitis	3/35 (8.6)	3/26 (12)	0/9 (0)	
Septic arthritis	2/35 (5.7)	2/26 (7.7)	0/9 (0)	
Endocarditis	1/35 (2.9)	1/26 (3.8)	0/9 (0)	
Endophthalmia	1/35 (2.9)	1/26 (3.8)	0/9 (0)	
None	28/35 (80)	19/26 (73)	9/9 (100)	
Bacterial co-infection	8/35 (23)	5/26 (19)	3/9 (33)	0.4
Invasive fungal co-infection	7/35 (20)	4/26 (15)	3/9 (33)	0.3
First-line antifungal treatment				0.5
Echinocandin monotherapy	25/35 (71)	19/26 (73)	6/9 (67)	
Fluconazole monotherapy	3/35 (8.6)	3/26 (12)	0/9 (0)	
Voriconazole monotherapy	2/35 (5.7)	1/26 (3.8)	1/9 (11)	
Combination therapy	2/35 (5.7)	1/26 (3.8)	1/9 (11)	
No antifungals	3/35 (8.6)	2/26 (7.7)	1/9 (11)	
Median length of hospital stay, d (IQR)	15 (7–31)	12 (6–23)	55 (22–64)	0.005
Recurrence	5/30 (17)	5/22 (23)	0/8 (0)	0.3
90-d all-cause mortality	1/30 (3.3)	0/22 (0)	1/8 (13)	0.3

*Values are no. (%) except as indicated. Data reported through **Reseau de Surveillance des Infections Fongiques invasives** (RESSIF**) and** Surveillance des Infections Fongiques Invasives **(**SINFONI). Denominators lower than the N/n value for each category indicate missing data. IDU, injecting drug user; IFD, invasive fungal disease; IQR, interquartile range. †Determined by Wilcoxon rank-sum test or Fisher exact test. ‡Other underlying conditions were hematologic malignancy (n = 1), solid organ malignancy (n = 1), premature neonate (n = 1), and severe malnutrition (n = 1).

Of the 35 patients in our study, 26 (74.2%) were IDUs and 20 had no other risk factor for IFD. Among the other usual risk factors for IFD, the presence of a central vascular catheter (n = 9) and ICU admission at diagnosis (n = 7) were the most frequent. Univariate analysis showed that those risk factors were significantly less frequent in IDU patients (p<0.001 by Fisher exact test). One patient was a premature neonate. We found deep-seated *W. anomalus* infections or secondary localizations with (n = 3) or without (n = 4) fungemia in 7 patients, all of whom were IDUs: 3 spondylodiscitis, 2 septic arthritis (wrist and knee), 1 endocarditis, and 1 endophthalmitis. We identified concurrent bacterial infections in 8 and fungal infection in 7 cases. In contrast to non-IDU cases, most *W. anomalus* infections in the IDU group were community acquired (p = 0.039 by Fisher exact test). Recurrent episodes occurred in 5 IDU patients: 4 experienced 3 episodes each, and 1 had 2 episodes. The overall 3-month mortality rate was low (1/30 deaths, 3.3%); we observed no significant differences between IDU and non-IDU groups.

### IDU Characteristics

We collected data on injected products for 23/26 IDU patients. Most (n = 16 [69.6%]) patients were multiple drug injectors. Heroin and cocaine were the most commonly injected substances (n = 13 each), followed by buprenorphine (n = 11), other opioids (n = 7), amphetamines (n = 4), and methylphenidate (n = 4). No single substance was common to all cases. In addition, 14 (60.9%) patients reported a history of alcohol abuse. A total of 21 patients documented their housing status; 13 (61.9%) were either homeless (n = 10) or living in precarious conditions (2 in caravans and 1 in a social hostel), whereas 8 (38.1%) resided in private housing.

### *W. anomalus* Isolates

**Among the 47 reported episodes of *W. anomalus* IFD, 36 isolates were available for subsequent analyses. We recovered multiple isolates from recurrent episodes in 5 patients. Of note, 1 isolate had been obtained from the cotton a patient had used to filter injected drugs; it had been collected concurrently with a *W. anomalus* fungemia. All identifications provided by MALDI-ToF mass spectrometry and sequencing methods were concordant. In addition, we included 19 *W. anomalus* isolates collected during 2004–2024, outside the RESSIF and SINFONI surveillance programs (involved or not in IFD), and the NRRL Y3668 reference strain in subsequent genotypic analyses, resulting in a total of 56 isolates.**

### STR Genotyping

**Figure 1:**
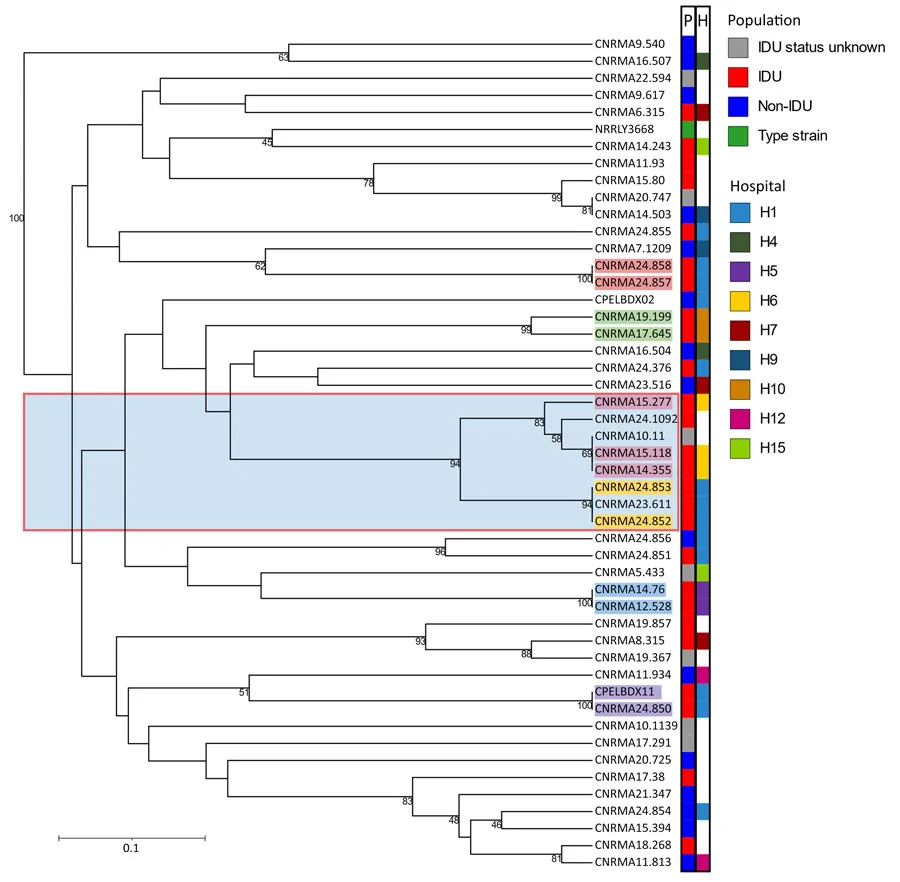
*Wickerhamomyces anomalus* isolates in study of invasive *W. anomalus* infections among injecting drug users, France, 2012–2024. Dendrogram was created by of 49 *W. anomalus* isolates genotyped using a 12-marker short tandem repeat approach. The P column indicates the IDU status of the patient from whom each isolate originated. The H column denotes hospital of origin for hospitals contributing >2 isolates. Blue shaded area indicates cluster of 7 IDU-associated isolates. Matching colored shading on isolate names indicates those isolates belong to the same patient. Numbers at nodes indicate bootstrap values. Scale bar represents the Bruvo genetic distance (short tandem repeat differences). IDU, injecting drug use.

**We subjected 49 of the 56 isolates to STR genotyping: 26 originating from IDU patients, 15 from non-IDU patients, and 7 from patients with unknown IDU status or environmental sources, as well as the reference strain. Analysis revealed 41 unique STR profiles identified across both IDU and non-IDU groups (** Figure 1 **). We observed no distinct genetic cluster among all IDU isolates. However, 8 isolates, including 7 from 4 IDU patients across different hospitals, formed a small cluster with limited variations in repeat numbers. Furthermore, 3 genotypes were shared across different patients: 1 was found in 2 IDU patients from the same hospital (isolates CNRMA23.611/CNRMA24.852, hospital H1), and the other 2 in patients who differed in time of infection, geographic location, and IDU status (isolates CNRMA20.747/CNRMA14.503 and CNRMA9.1011/CNRMA15.118). In addition, an IDU patient’s blood culture isolate (isolate CNRMA24.858) and the isolate recovered from the cotton used by the same patient to filter drugs, collected concomitantly with the fungemia (isolate CNRMA24.857) shared the same genotype. Finally, regarding recurrent episodes, all isolates were genotypically identical (isolates CNRMA12.528/CNRMA14.76, CNRMA24.850/CPELBDX11, and CNRMA24.853/CNRMA24.852) or showed a minor repeat number variation at a single locus (isolates CNRMA15.118/CNRMA15.277), except for 1 patient who had 2 *W. anomalus* isolates with distant STR profiles (isolates CNRMA19.199/CNRMA17.645).**

### Whole-Genome Sequencing Analysis

**Figure 2:**
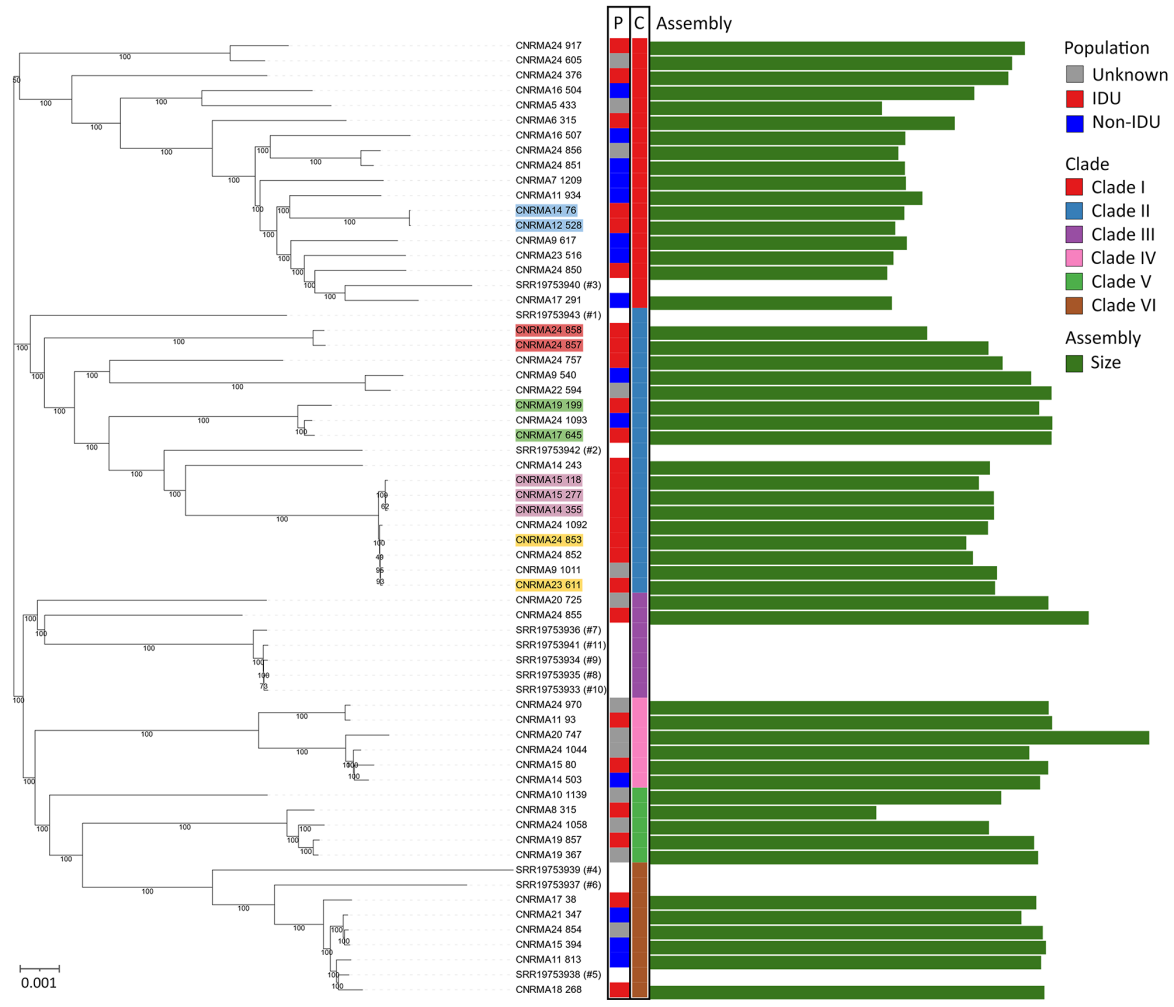
Phylogenetic tree of *Wickerhamomyces anomalus* isolates in study of invasive *W. anomalus* infections among injecting drug users, France, 2012–2024. Tree is based on genomewide SNPs from 53 *W. anomalus* isolates collected in the study and 11 publicly available genomes (isolate names starting with SRR) and was developed by neighbor-joining method. The P column indicates the IDU status of the patient from whom each isolate originated. The C column denotes phylogenetic clade. Matching colored shading on isolate names indicates those isolates belong to the same patient. Green bars represent assembled genome size of 13.9–29.7 Mb. Variation in assembly size reflects levels of genomic heterozygosity: isolates with extensive loss of heterozygosity (clade 1) have smaller assemblies caused by haplotype collapse, whereas highly heterozygous isolates have larger assemblies with uncollapsed haplotypes. Numbers at nodes indicate bootstrap values. Scale bar represents genetic distance (proportion of differing SNPs). IDU, injecting drug use; SNP, single-nucleotide polymorphism.

**Figure 3:**
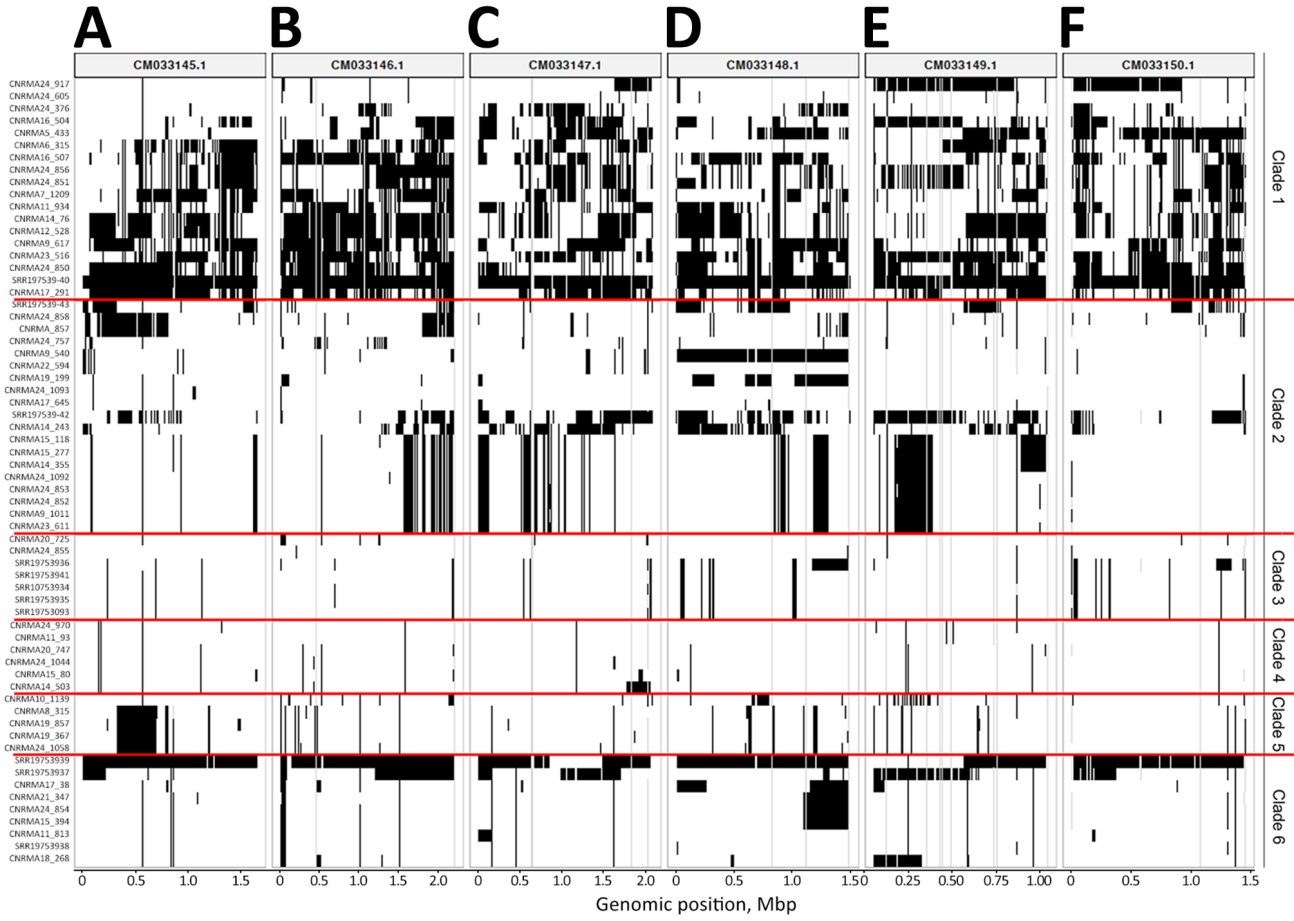
Genomewide loss of heterozygosity distribution across 6 chromosomes of *Wickerhamomyces anomalus* fungi in study of invasive *W. anomalus* infections among injecting drug users, France, 2012–2024. We analyzed 53 *W. anomalus* isolates collected in this study and 11 publicly available genomes, grouped by phylogenetic clade. Chromosomes are CM033145.1 (A), CM033146.1 (B), CM033147.1 (C), CM033148.1 (D), CM033149.1 (E), and CM033150.1 (F). Red lines indicate clades. Black indicates loss of heterozygosity (LOH); gray, insufficient variant coverage; white (no color), heterozygosity. LOH patterns vary extensively within and between clades. Clade 1 (top) exhibits high levels of LOH and substantial variation between isolates. Other clades show more moderate and heterogeneous LOH distributions. Some isolates within a clade share similar patterns; others display distinct profiles.

**Whole-genome sequencing (WGS) analysis of 53 of the 56 isolates from this study, together with 11 publicly available genomes (**Appendix** Table 1), yielded an alignment of 391,733 SNPs and revealed 6 main clades (**Figure 2**). Across all 64 genomes, pairwise distances were 742–346,637 (mean 208,986) SNPs. Within-patient pair ranges were 742–15,296 SNPs and all clustered together in the phylogeny, including the 2 that appeared distant by STR (isolates CNRMA17.645 and CNRMA19.199). The STR locus implicated in this discordance, namely M3b, lies within an LOH region on chromosome CM033148.1 (positions 1,446,243–1,447,304) (**Figure 3**) in CNRMA19.199, explaining the discrepancy. Genetic diversity indices computed on 16,935 LD-pruned SNPs across 52 isolates (excluding LOH-affected isolates) confirmed substantial allelic diversity (Simpson λ = 0.98; Nei unbiased gene diversity Hexp = 0.38 + 0.08), with moderate differentiation between phylogenetic clades (global FST = 0.16). Population structure using ADMIXTURE revealed 7 genetic clusters broadly concordant with the 6 phylogenetic clades: 2 clades showed exclusive cluster assignment, whereas the remaining 4 displayed admixed profiles (**Appendix Figure 3, pa**nel A). Isolates from the same city consistently belonged to distinct clusters, providing no evidence for city-level population structure (**Appendix Figure 3, pa**nel B).**

**De novo assemblies were 12–30 Mb, consistent with results from public datasets for *W. anomalus* from the National Center for Biotechnology Information in which 26 genomes spanned 12.72–26.55 Mb (**https://www.ncbi.nlm.nih.gov/datasets/genome/?taxon=4927**; accessed 2025 Sep 16). Isolates with the smallest assemblies clustered within a subclade enriched for LOH regions (**Figures 2, 3**), suggesting that expanded LOH promotes haplotig coalescence and yields more compact assemblies. QUAST (**https://quast.sourceforge.net**) further showed that assembly size increases with the duplication ratio (**Appendix Figure 4), **consistent with retention of haplotigs or unpurged duplicated segments, or both. We observed similar patterns in several of the assemblies, indicating that larger assembly sizes often reflect duplicated content rather than intrinsically larger genomes. Finally, VAF profiles (**Appendix Figure 5) **showed shifts compatible with segmental aneuploidy in some genomic regions,** **al**though not fully supported by read depth. 

## Discussion

This retrospective multicentric nationwide study, conducted over a 13-year period, provides new insights on *W. anomalus* IFD. Of note, IDU emerged as a major risk factor, representing the sole underlying condition in 20 (57.1%) of 35 cases **(**Table**)**. An additional 6 cases occurred in IDU patients with other predisposing factors for IFD. Furthermore, a marked increase in *W. anomalus* IFD has been observed since 2020, primarily driven by IDU-related infections, as 14 (53.8%) of 26 cases occurred after 2020. In contrast to previous reports, we observed no hospital-based outbreaks in ICU or neonatal settings; of note, only 1 case was documented in a premature neonate. All but 1 non-IDU patients had classical risk factors for yeast IFD; **that** patient was homeless and exhibited behaviors suggestive of IDU, although we could not definitively confirm whether that was correct.

Although IDU is a well-recognized risk factor for invasive candidiasis (*32**,**33*), only 1 case of *W. anomalus* infection in an IDU had been reported in the literature (*34*) before a study published in 2025 that reported 7 cases and demonstrated a notable overrepresentation of *W. anomalus* yeast in IDU-associated infections (*18*). However, a US study on fungemia among IDU patients did not report that association (*35*), suggesting that the emergence of *W. anomalus* infections may reflect local transmission dynamics, potentially linked to specific drug products, injection practices, or environmental exposures. **Of interest, in the study we report, a *W. anomalus* isolate was recovered from the cotton a patient had used to filter injected drugs, which was collected concurrently with a blood culture that tested positive for *W. anomalus*. Genotyping analysis revealed that both *W. anomalus* isolates shared an identical STR genotype. The patient was otherwise colonized by *Nakaseomyces glabratus* (**formerly ***Candida glabrata*) yeast. Taken together, those findings strongly suggest direct inoculation via a contaminated drug preparation, rather than skin contamination. However, we identified no specific drug product as associated with the risk for *W. anomalus* IFD in our cohort. In addition, STR genotyping and WGS analyses did not reveal any specific cluster among IDU-associated cases, arguing against contamination from a single source or a unique product over the 13-year study period. We did not identify consistent environmental exposure either; patients had diverse housing situations across 15 cities. Nevertheless, 8 (30.8%) of 26 patients originated from the same city, and 2 patients shared an identical STR genotype within a 6-month interval, suggesting possible local transmission or exposure. Finally, injection practices themselves may contribute to the transmission risk for *W. anomalus*. Previous reports have linked fungemia in IDU populations to the use of contaminated lemon juice for diluting brown heroin** (*36*)**.** **Given that *W. anomalus* yeast is commonly found in water sources, food products, and fermented beverages such as wine and beer, exposure could plausibly occur through the use of nonsterile diluents. However, further environmental investigations and prospective practices surveys are warranted to substantiate our hypothesis.**

**Most (n = 25) *W. anomalus* IFD patients were treated with echinocandins either as monotherapy or in combination with other antifungals (n = 2). Remarkably, the overall 3-month mortality rate remained low (3.3%, n = 1/30) across both IDU and non-IDU groups, even in instances in which antifungal therapy was absent (n = 3) or limited to fluconazole (n = 3), despite elevated minimal inhibitory concentrations to fluconazole** (*37*) **(**Appendix Table 1). **That observation may suggest that *W. anomalus* exhibits lower virulence than other yeast species commonly implicated in fungemia.**

**MALDI-TOF mass spectrometry using commercial databases appears to be an efficient method for accurate species-level identification of *W. anomalus* in clinical practice. In addition, both STR and WGS methods are suitable approaches for *W. anomalus* genotyping, yielding overall concordant results.** However, *W. anomalus* is a diploid heterothallic species, and our WGS analysis suggests that LOH events are frequently happening among this species. Genome assemblies of 12–30 Mb in our study match those of public data; smaller assemblies linked to LOH and larger ones to unpurged duplications (Appendix Figure 4). In that context, WGS SNP analysis might have overestimated the number of SNPs, suggesting that STR genotyping is a more reliable approach for assessing isolates’ relatedness and explaining the minor discordances observed between the 2 methods. **LOH events have been shown to enable rapid adaptation to both host environments and antifungal exposure** (*38*); they **have also been reported to constitute a major evolutionary mechanism driving divergence within *C. albicans* populations** (*39*). **LOH can lead to loss-of-function alleles with notable phenotypic variations.** An example is in C. albicans clade I isolates, which carry a heterozygous LOF mutation in FUR1 conferring elevated intrinsic resistance to 5-fluorocytosine (*40*); once rendered homozygous by LOH, the mutation results in complete resistance to the drug. Four isolates exhibited resistance to 5-fluorocytosine in vitro (Appendix Table 1), but none showed evidence of LOH affecting the *FUR1* homologue. Nevertheless, we observed an interesting LOH event in 2 isolates recovered from the same patient at different times (isolates CNRMA17.645 in 2017 and CNRMA19.199 in 2019). In such pathogen, LOH might represent a more immediate adaptive response to environmental stress than sexual recombination. Additional analysis could be useful to determine precise chromosomic location of LOH and eventually **loss-of-function** related.

**Data collection issues affected our study. Reported IDU status and drugs injected might not reflect the patient’s situation at the time of the IFD episode.** Similarly, because neither RESSIF nor SINFONI was specifically designed to capture **IDU infections**, participating centers might have missed information on injected products. In addition, the relatively limited number of cases and the imbalance between the numbers of IDU and non-IDU patients restrict the possibility of assessing confounding risk factors between the 2 groups through multivariate analysis.

**In conclusion, this nationwide study in France** highlights the increased risk for W. anomalus IFD among persons who use injected drugs; we observed a rise in cases **since 2021. Our findings strongly suggest an exogenous source of infection, potentially linked to environmental exposure or unsafe injection practices,** and emphasize the importance of targeted preventive measures within IDU populations.

Appendix Additional information about invasive *Wickerhamomyces anomalus* infections among injecting drug users, France, 2012–2024.

## References

[1] Kurtzman CP. Phylogeny of the ascomycetous yeasts and the renaming of *Pichia anomala* to *Wickerhamomyces anomalus.* Antonie van Leeuwenhoek. 2011;99:13–23. 10.1007/s10482-010-9505-6 20838888

[2] Passoth V, Fredlund E, Druvefors UÃ, Schnürer J. Biotechnology, physiology, and genetics of the yeast *Pichia anomala.* FEMS Yeast Res. 2006;6:3–13. 10.1111/j.1567-1364.2005.00004.x 16423066

[3] López-Enríquez L, Vila-Crespo J, Rodríguez-Nogales JM, Fernández-Fernández E, Ruipérez V. Modulation of the aromatic profile of Verdejo wine through sequential inoculation of *Wickerhamomyces anomalus* and *Saccharomyces cerevisiae.* Fermentation (Basel). 2023;9:977. 10.3390/fermentation9110977

[4] Bretagne S, Sitbon K, Desnos-Ollivier M, Garcia-Hermoso D, Letscher-Bru V, Cassaing S, et al.; French Mycoses Study Group. Active surveillance program to increase awareness on invasive fungal diseases: the French RESSIF Network (2012 to 2018). mBio. 2022;13:e0092022. 10.1128/mbio.00920-22 35499498 PMC9239099

[5] Díaz-García J, Mesquida A, Sánchez-Carrillo C, Reigadas E, Muñoz P, Escribano P, et al. Monitoring the epidemiology and antifungal resistance of yeasts causing fungemia in a tertiary care hospital in Madrid, Spain: any relevant changes in the last 13 years? Antimicrob Agents Chemother. 2021;65:e01827–20. 10.1128/AAC.01827-20 33468487 PMC8097463

[6] Song Y, Chen X, Yan Y, Wan Z, Liu W, Li R. Prevalence and antifungal susceptibility of pathogenic yeasts in China: a 10-year retrospective study in a teaching hospital. Front Microbiol. 2020;11:1401. 10.3389/fmicb.2020.01401 32719663 PMC7347963

[7] Lin HC, Lin HY, Su BH, Ho MW, Ho CM, Lee CY, et al. Reporting an outbreak of *Candida pelliculosa* fungemia in a neonatal intensive care unit. J Microbiol Immunol Infect. 2013;46:456–62. 10.1016/j.jmii.2012.07.013 23050985

[8] Pasqualotto AC, Sukiennik TCT, Severo LC, de Amorim CS, Colombo AL. An outbreak of *Pichia anomala* fungemia in a Brazilian pediatric intensive care unit. Infect Control Hosp Epidemiol. 2005;26:553–8. 10.1086/502583 16018431

[9] Murphy N, Buchanan CR, Damjanovic V, Whitaker R, Hart CA, Cooke RWI. Infection and colonisation of neonates by *Hansenula anomala.* Lancet. 1986;1:291–3. 10.1016/S0140-6736(86)90827-5 2868164

[10] Chakrabarti A, Singh K, Narang A, Singhi S, Batra R, Rao KLN, et al. Outbreak of *Pichia anomala* infection in the pediatric service of a tertiary-care center in northern India. J Clin Microbiol. 2001;39:1702–6. 10.1128/JCM.39.5.1702-1706.2001 11325977 PMC88012

[11] Yang Y, Wu W, Ding L, Yang L, Su J, Wu B. Two different clones of *Candida pelliculosa* bloodstream infection in a tertiary neonatal intensive care unit. J Infect Dev Ctries. 2021;15:870–6. 10.3855/jidc.12103 34242199

[12] da Silva CM, de Carvalho Parahym AMR, Leão MPC, de Oliveira NT, de Jesus Machado Amorim R, Neves RP. Fungemia by *Candida pelliculosa* (*Pichia anomala*) in a neonatal intensive care unit: a possible clonal origin. Mycopathologia. 2013;175:175–9. 10.1007/s11046-012-9605-0 23232762

[13] Jung J, Moon YS, Yoo JA, Lim JH, Jeong J, Jun JB. Investigation of a nosocomial outbreak of fungemia caused by *Candida pelliculosa* (*Pichia anomala*) in a Korean tertiary care center. J Microbiol Immunol Infect. 2018;51:794–801. 10.1016/j.jmii.2017.05.005 28779880

[14] Zhang L, Xiao M, Arastehfar A, Ilkit M, Zou J, Deng Y, et al. Investigation of the emerging nosocomial *Wickerhamomyces anomalus* infections at a Chinese tertiary teaching hospital and a systemic review: clinical manifestations, risk factors, treatment, outcomes, and anti-fungal susceptibility. Front Microbiol. 2021;12:744502. 10.3389/fmicb.2021.744502 34690991 PMC8527005

[15] Thuler LCS, Faivichenco S, Velasco E, Martins CA, Nascimento CRG, Castilho IAMA. Fungaemia caused by *Hansenula anomala*—an outbreak in a cancer hospital. Mycoses. 1997;40:193–6. 10.1111/j.1439-0507.1997.tb00213.x 9476487

[16] Evren K, Akçay E, Yücel M, Bal AZ, Erdinç FŞ, Dinç B, et al. A case of peritonitis caused by *Wickerhamomyces anomalus* (*Candida pelliculosa*) related to peritoneal dialysis [in Turkish]. Mikrobiyol Bul. 2021;55:665–72. 10.5578/mb.20219718 34666666

[17] Mehta V, Mohanty A, Meena S, Rahul JS, Uttam Kumar N, Chattopadhyay D, et al. *Wickerhamomyces anomalous*: a rare cause of fungemia causing febrile neutropenia in acute lymphoblastic leukemia. Case Rep Infect Dis. 2020;2020:8847853. 10.1155/2020/8847853 33457028 PMC7785382

[18] Paccoud O, Lortholary O, Imbert S, Letscher-Bru V, Boukris-Sitbon K, Obadia T, et al.; French Mycoses Study Group. Yeast fungaemia among injection drug users in France (2012–2022): a cross-sectional observational study. Lancet Reg Health Eur. 2025;55:101365. 10.1016/j.lanepe.2025.101365 40950950 PMC12426822

[19] von Elm E, Altman DG, Egger M, Pocock SJ, Gøtzsche PC, Vandenbroucke JP; STROBE Initiative. Strengthening the Reporting of Observational Studies in Epidemiology (STROBE) statement: guidelines for reporting observational studies. BMJ. 2007;335:806–8. 10.1136/bmj.39335.541782.AD 17947786 PMC2034723

[20] Kurtzman CP, Robnett CJ. Identification and phylogeny of ascomycetous yeasts from analysis of nuclear large subunit (26S) ribosomal DNA partial sequences. Antonie van Leeuwenhoek. 1998;73:331–71. 10.1023/A:1001761008817 9850420

[21] Guinea J, Meletiadis J, Arikan-Akdagli S, Giske C, Muehlethaler K, Arendrup MC. Method for the determination of broth dilution minimum inhibitory concentrations of antifungal agents for yeasts. 2023 [cited 2025 Nov 19]. https://www.eucast.org/fileadmin/eucast/pdf/AFST/methodology/EUCAST_E.Def_7.4_Yeast_definitive_revised_2023.pdf

[22] Spruijtenburg B, Rudramurthy SM, Meijer EFJ, van Haren MHI, Kaur H, Chakrabarti A, et al. Application of novel short tandem repeat typing for *Wickerhamomyces anomalus* reveals simultaneous outbreaks within a single hospital. Microorganisms. 2023;11:1525. 10.3390/microorganisms11061525 37375027 PMC10303041

[23] Kamvar ZN, Tabima JF, Grünwald NJ. Poppr: an R package for genetic analysis of populations with clonal, partially clonal, and/or sexual reproduction. PeerJ. 2014;2:e281. 10.7717/peerj.281 24688859 PMC3961149

[24] Letunic I, Bork P. Interactive Tree Of Life (iTOL) v5: an online tool for phylogenetic tree display and annotation. Nucleic Acids Res. 2021;49(W1):W293–6. 10.1093/nar/gkab301 33885785 PMC8265157

[25] Ewels P, Magnusson M, Lundin S, Käller M. MultiQC: summarize analysis results for multiple tools and samples in a single report. Bioinformatics. 2016;32:3047–8. 10.1093/bioinformatics/btw354 27312411 PMC5039924

[26] Criscuolo A, Brisse S. AlienTrimmer: a tool to quickly and accurately trim off multiple short contaminant sequences from high-throughput sequencing reads. Genomics. 2013;102:500–6. 10.1016/j.ygeno.2013.07.011 23912058

[27] Knaus BJ, Grünwald NJ. vcfr: a package to manipulate and visualize variant call format data in R. Mol Ecol Resour. 2017;17:44–53. 10.1111/1755-0998.12549 27401132

[28] Paradis E, Claude J, Strimmer K. APE: Analyses of Phylogenetics and Evolution in R language. Bioinformatics. 2004;20:289–90. 10.1093/bioinformatics/btg412 14734327

[29] Prjibelski A, Antipov D, Meleshko D, Lapidus A, Korobeynikov A. Using SPAdes de novo assembler. Curr Protoc Bioinformatics. 2020;70:e102. 10.1002/cpbi.102 32559359

[30] Mikheenko A, Saveliev V, Hirsch P, Gurevich A. WebQUAST: online evaluation of genome assemblies. Nucleic Acids Res. 2023;51(W1):W601–6. 10.1093/nar/gkad406 37194696 PMC10320133

[31] Wickham H. Data analysis. In: ggplot2. Use R! 2nd ed. Cham (Switzerland): Springer International Publishing; 2016. p. 189–201.

[32] Poowanawittayakom N, Dutta A, Stock S, Touray S, Ellison RT III, Levitz SM. Reemergence of intravenous drug use as risk factor for candidemia, Massachusetts, USA. Emerg Infect Dis. 2018;24:631–7. 10.3201/eid2404.171807 29553923 PMC5875264

[33] Rossow JA, Gharpure R, Brennan J, Relan P, Williams SR, Vallabhaneni S, et al. Injection drug use-associated candidemia: incidence, clinical features, and outcomes, east Tennessee, 2014–2018. J Infect Dis. 2020;222(Suppl 5):S442–50. 10.1093/infdis/jiaa024 32877559 PMC8186399

[34] Nohinek B, Zee-Cheng CS, Barnes WG, Dall L, Gibbs HR. Infective endocarditis of a bicuspid aortic valve caused by *Hansenula anomala.* Am J Med. 1987;82:165–8. 10.1016/0002-9343(87)90400-1 3799679

[35] Zhang AY, Shrum S, Williams S, Petnic S, Nadle J, Johnston H, et al. The changing epidemiology of candidemia in the United States: injection drug use as an increasingly common risk factor-active surveillance in selected sites, United States, 2014–2017. Clin Infect Dis. 2020;71:1732–7. 10.1093/cid/ciz1061 31676903

[36] Miró JM, Puig de la Bellacasa J, Odds FC, Gill BK, Bisbe J, Gatell JM, et al. Systemic candidiasis in Spanish heroin addicts: a possible source of infection. J Infect Dis. 1987;156:857–8. 10.1093/infdis/156.5.857 3309077

[37] Luo Z, Ning Y, Xiao M, Guo D, Xu H, Liu Y, et al. High azole non-wild type rates and nosocomial microsatellite typing aggregation of *Wickerhamomyces anomalus* in China according to a 12-year multicenter surveillance study. J Antimicrob Chemother. 2025;80:1964–71. 10.1093/jac/dkaf156 40396695 PMC12209787

[38] Ford CB, Funt JM, Abbey D, Issi L, Guiducci C, Martinez DA, et al. The evolution of drug resistance in clinical isolates of *Candida albicans.* eLife. 2015;4:e00662. 10.7554/eLife.00662 25646566 PMC4383195

[39] Wang JM, Bennett RJ, Anderson MZ. The genome of the human pathogen *Candida albicans* is shaped by mutation and cryptic sexual recombination. mBio. 2018;9:e01205–18. 10.1128/mBio.01205-18 30228236 PMC6143739

[40] Dodgson AR, Dodgson KJ, Pujol C, Pfaller MA, Soll DR. Clade-specific flucytosine resistance is due to a single nucleotide change in the *FUR1* gene of *Candida albicans.* Antimicrob Agents Chemother. 2004;48:2223–7. 10.1128/AAC.48.6.2223-2227.2004 15155225 PMC415630

