# Supplementary material for: Invasive Wickerhamomyces anomalus Infections among Injecting Drug Users, France, 2012–2024
Source: Emerg Infect Dis. 2026 Sep;32(9):1452–62. doi: 10.3201/eid3209.251817 (PMC13555705; doi:10.3201/eid3209.251817)
Supplement: Supplementary file 1 [file 25-1817-Techapp-s1.pdf]

Article DOI: https://doi.org/10.3201/eid3209.251817

EID cannot ensure accessibility for supplementary materials supplied by authors. Readers who have difficulty accessing supplementary content should contact the authors for assistance.

Invasive *Wickerhamomyces anomalus* Infections among Injecting Drug Users, France, 2012–2024

Appendix

Detailed Methods for Variant Calling and Population Genetic Analyses

Variant Calling

Reads were aligned to the *W. anomalus* KG16 reference genome (GCA_019321675.1) using BWA-MEM (v0.7.17-r1188) with default parameters (*1*). The -R option was used to set sample-specific read groups following GATK recommendations. SAM files were converted to BAM, sorted, and indexed using samtools (v1.16) (*2*). PCR duplicates were identified and marked with MarkDuplicates (Picard v2.23.3) (*3*). Variant calling was performed using the Genome Analysis Toolkit (GATK; v4.6.0.0) with the HaplotypeCaller algorithm and the -ERC GVCF option (*4*). All GVCF files were combined into a single genomic database using GATK GenomicsDBImport, and joint genotyping was performed with GATK GenotypeGVCFs. Variants were filtered with GATK VariantFiltration using the following thresholds: QD <2.0 (quality by depth), QUAL <100.0 (variant confidence), FS >60.0 (Fisher strand bias), MQ <40.0 (mapping quality), ReadPosRankSum <-8.0 (read position rank sum), AF <0.15, and AF >0.90 (Allele Frequency). GATK SelectVariants was used to generate a VCF containing only SNPs passing these filters. To further restrict analyses to high-confidence variants, the resulting VCF was filtered to retain only biallelic SNPs using bcftools[5] view -m2 -M2 -v snps and sites with “spanning deletions” (ALT = *) were removed (-e 'ALT~”*”').

Population Genomic Analyses

Per-isolate proportion of heterozygous SNP genotypes was computed across all variants sites as a proxy for genome-wide loss of heterozygosity (LOH). Twelve isolates displaying

heterozygosity rates below 0.20 (consistent with LOH), were excluded. The remaining 52 isolates were processed with PLINK version 2.0.0-a.6.11 (–maf 0.05,–geno 0.10 and –mind 0.20) and LD-pruned (–indep-pairwise 50 10 0.3), retaining 16,935 SNPs across 52 isolates for downstream analysis (*5*).

Genetic diversity and differentiation was assessed in R version 4.3.2 using the vcfR package (version 1.15.0), popper (v2.9.6) and hierfstat (v0.5–11) packages (*6*). Simpson's diversity index (λ) was computed using the the poppr package (*7*). Nei's unbiased gene diversity (Hexp) and global genetic differentiation between phylogenetic clades (FST) were computed using the hierfstat package (*8*,*9*).

Population structure was inferred using ADMIXTURE version 1.3.1 (*10*). The LD-pruned PLINK binary files were used as input files. Models were run for K = 1 to 10 with 10-fold cross-validation (–cv. = 10). K = 7 was selected based on the minimum cross-validation error (CV = 0.23, 10-fold). Results were visualized using ggplot2 package.

Loss of Heterozygosity Map

For each sample and genomic bin (size 3,000 bp), the proportion of heterozygous sites was calculated based on variant allele frequency (VAF): positions with VAF <0.2 were called "homozygous reference," VAF >0.8 as "homozygous alternative," and intermediate values as "heterozygous." Bins with fewer than 20 variants were ignored; bins with less than 5% heterozygous sites were classified as LOH. The distribution of LOH regions across chromosomes and individuals was visualized as LOH state maps, with bins classified as "LOH," "heterozygous" or "NA" and plotted as colored tiles for each sample and chromosome.

References

1. Li H. Aligning sequence reads, clone sequences and assembly contigs with BWA-MEM. 2013 [cited 2025 Nov 19]. http://arxiv.org/abs/1303.3997 doi:10.48550/arXiv.1303.3997

2. Danecek P, Bonfield JK, Liddle J, Marshall J, Ohan V, Pollard MO, et al. Twelve years of SAMtools and BCFtools. Gigascience. 2021;10:giab008. PubMed https://doi.org/10.1093/gigascience/giab008

3. Broad Institute. Picard Toolkit [cited 2025 Nov 19]. http://broadinstitute.github.io/picard

4. Van der Auwera GA, Carneiro MO, Hartl C, Poplin R, Del Angel G, Levy-Moonshine A, et al. From FastQ data to high confidence variant calls: the Genome Analysis Toolkit best practices pipeline. Curr Protoc Bioinformatics. 2013;43:10.1, 33. PubMed https://doi.org/10.1002/0471250953.bi1110s43

5. Chang CC, Chow CC, Tellier LC, Vattikuti S, Purcell SM, Lee JJ. Second-generation PLINK: rising to the challenge of larger and richer datasets. GigaScience. 2015;4:s13742-015-0047-8. https://doi.org/10.1186/s13742-015-0047-8

6. Goudet J. HIERFSTAT, a package for R to compute and test hierarchical *F* -statistics. Mol Ecol Notes. 2005;5:184–6. https://doi.org/10.1111/j.1471-8286.2004.00828.x

7. Simpson EH. Measurement of diversity. Nature. 1949;163:688. https://doi.org/10.1038/163688a0

8. Nei M. Estimation of average heterozygosity and genetic distance from a small number of individuals. Genetics. 1978;89:583–90. PubMed https://doi.org/10.1093/genetics/89.3.583

9. Weir BS, Cockerham CC. Estimating F-statistics for the analysis of population structure. Evolution. 1984;38:1358–70. PubMed https://doi.org/10.2307/2408641

10. Alexander DH, Novembre J, Lange K. Fast model-based estimation of ancestry in unrelated individuals. Genome Res. 2009;19:1655–64. PubMed https://doi.org/10.1101/gr.094052.109

Appendix Table. Metadata of isolates included in the WGS analyses*

WGS accession		Strain collection				MIC (mg/L)										
number	Strain number	Sample type	Country	City	Year	AMB	FC	FLU	ITZ	VRC	POS	ISA	ANI	MICA	CAS	Reference
SRR19753933	08-32-01-02	Blood culture	India	Chandigarh	2019	0.125	NA	1	0.25	0.5	NA	0.06	NA	0.015	NA	10.3390/microorganisms11061525
SRR19753936	08-32-01-05	Blood culture	India	Chandigarh	2019	0.125	NA	4	0.25	0.125	NA	0.06	NA	0.03	NA	10.3390/microorganisms11061525
SRR19753934	08-32-01-07	Blood culture	India	Chandigarh	2019	0.25	NA	1	0.25	0.125	NA	0.06	NA	<0.008	NA	10.3390/microorganisms11061525
SRR19753935	08-32-01-14	Blood culture	India	Chandigarh	2019	0.125	NA	1	0.125	0.125	NA	0.06	NA	<0.008	NA	10.3390/microorganisms11061525
SRR19753937	08-32-01-15	Blood culture	India	Chandigarh	2019	0.25	NA	2	0.25	0.125	NA	0.06	NA	<0.008	NA	10.3390/microorganisms11061525
SRR19753939	08-32-01-29	Blood culture	India	Chandigarh	2019	0.125	NA	4	0.25	0.125	NA	0.06	NA	<0.008	NA	10.3390/microorganisms11061525
SRR19753941	08-32-01-30	Blood culture	India	Chandigarh	2019	0.125	NA	1	0.125	0.125	NA	0.06	NA	<0.008	NA	10.3390/microorganisms11061525
SRR19753943	10-03-01-56	Blood culture	Qatar	Doha	2019	0.125	NA	8	0.5	0.25	NA	0.06	0.015	0.015	NA	10.3390/microorganisms11061525
SRR19753938	10-04-08-74	Blood culture	The Netherlands	Nijmegen	2019	0.25	NA	2	0.06	0.125	NA	NA	<0.008	0.015	NA	10.3390/microorganisms11061525
SRR19753940	10-09-05-80	Blood culture	Italy	Pavia	1984	0.015	NA	2	0.015	0.06	NA	0.03	0.008	<0.008	NA	10.3390/microorganisms11061525
SRR19753942	10-11-09-22	Blood culture	The Netherlands	Nijmegen	2018	0.06	NA	16	0.06	0.125	NA	0.06	0.008	<0.008	NA	10.3390/microorganisms11061525
ERR15308104	CNRMA 10.1139	Blood culture	France	Paris	2010	0.125	0.125	2	NA	0.125	0.5	NA	0.015	0.03	0.06	This study
ERR15308105	CNRMA 11.813	Blood culture	France	Paris	2011	0.06	0.125	2	NA	0.125	0.25	NA	0.03	0.03	0.03	This study
ERR15308106	CNRMA 11.93	Blood culture	France	Paris	2010	0.125	0.125	4	NA	0.125	0.25	NA	0.03	0.015	0.06	This study
ERR15308107	CNRMA 11.934	Blood culture	France	Paris	2011	0.125	0.125	4	NA	0.125	0.25	NA	0.03	0.03	0.06	This study
ERR15308108	CNRMA 12.528	Blood culture	France	Paris	2012	0.125	32	2	NA	0.125	0.25	NA	0.06	0.03	0.06	This study
ERR15308109	CNRMA 14.243	Blood culture	France	Nice	2014	0.03	0.06	0.5	NA	0.06	0.5	NA	NA	0.03	0.06	This study
ERR15308110	CNRMA 14.355	Blood culture	France	Besancon	2014	0.125	0.06	4	NA	0.125	0.125	NA	NA	0.03	0.06	This study
ERR15308111	CNRMA 14.503	Blood culture	France	Paris	2014	0.06	0.06	2	NA	0.03	0.25	NA	NA	0.03	0.06	This study
ERR15308112	CNRMA 14.76	Blood culture	France	Paris	2014	0.06	0.125	2	NA	0.25	0.125	NA	0.03	0.015	0.03	This study
ERR15308113	CNRMA 15.118	Blood culture	France	Besancon	2014	0.06	0.06	4	NA	0.125	1	NA	NA	0.015	0.03	This study
ERR15308114	CNRMA 15.277	Blood culture	France	Besancon	2015	0.03	0.06	2	NA	0.06	0.125	NA	NA	0.008	0.015	This study
ERR15308115	CNRMA 15.394	Blood culture	France	Rennes	2014	0.015	0.250	0.5	NA	0.03	0.125	NA	NA	0.125	0.125	This study
ERR15308116	CNRMA 15.80	Blood culture	France	Tours	2013	0.03	0.06	2	NA	0.06	0.06	NA	NA	0.015	0.03	This study
ERR15308117	CNRMA 16.504	Eye	France	Paris	2016	0.125	0.06	2	NA	0.06	0.25	NA	NA	0.015	0.015	This study
ERR15308118	CNRMA 16.507	Eye	France	Paris	2016	0.06	0.06	2	NA	0.06	0.125	NA	NA	0.015	0.03	This study
ERR15308119	CNRMA 17.291	Blood culture	France	Paris	2017	0.03	0.06	1	NA	0.125	0.125	0.06	NA	0.015	0.03	This study
ERR16834401	CNRMA 17.38	Bone	France	Toulouse	2016	0.03	0.06	8	NA	0.25	0.5	0.06	NA	0.015	0.03	This study
ERR15308120	CNRMA 17.645	Blood culture	France	Dijon	2017	0.03	0.06	2	NA	0.06	0.25	0.03	NA	0.015	0.03	This study
ERR15308121	CNRMA 18.268	Blood culture	France	Strasbourg	2017	0.03	0.06	2	NA	0.125	0.25	0.03	NA	0.015	0.03	This study
ERR15308122	CNRMA 19.199	Blood culture	France	Dijon	2018	0.125	0.06	4	NA	0.25	0.5	0.125	NA	0.015	0.06	This study
ERR15308123	CNRMA 19.367	Skin	France	Reunion	2019	0.03	0.125	1	NA	0.06	0.125	0.06	NA	0.015	0.03	This study
ERR15308124	CNRMA 19.857	Blood culture	France	Caen	2019	0.06	0.06	2	NA	0.125	0.25	0.06	NA	0.015	0.015	This study
ERR15308125	CNRMA 20.725	Blood culture	France	Poitiers	2020	0.06	0.06	2	NA	0.06	0.125	0.06	NA	0.015	0.015	This study
ERR15308126	CNRMA 20.747	Blood culture	France	Valence	2020	0.06	0.125	2	NA	0.06	0.125	0.06	NA	0.015	0.03	This study
ERR15308127	CNRMA 21.347	Blood culture	France	Paris	2021	0.03	0.125	2	NA	0.125	0.25	0.06	NA	0.015	0.03	This study
ERR15308128	CNRMA 22.594	Blood culture	France	Perigueux	2022	0.03	0.125	2	NA	0.125	0.25	0.03	NA	0.015	0.03	This study
ERR15308129	CNRMA 23.516	Blood culture	France	Paris	2023	0.25	0.125	4	NA	0.125	0.125	0.125	NA	0.03	0.03	This study
ERR15308130	CNRMA 23.611	Blood culture	France	Bordeaux	2023	0.5	0.06	4	NA	0.25	0.5	0.03	NA	0.03	0.03	This study
ERR15308131	CNRMA 24.1044	Blood culture	France	Amiens	2024	0.5	0.125	2	NA	0.06	0.125	0.03	NA	0.03	0.015	This study
ERR15308132	CNRMA 24.1058	Blood culture	France	Antibes	2024	0.5	0.125	2	NA	0.06	0.25	0.125	NA	0.06	0.03	This study
ERR15308133	CNRMA 24.1092	Blood culture	France	Toulon	2024	0.5	0.125	2	NA	0.125	0.5	0.06	NA	0.03	0.015	This study
ERR15308134	CNRMA 24.1093	Blood culture	France	Angers	2024	NA	NA	NA	NA	NA	NA	NA	NA	NA	NA	This study
ERR15308135	CNRMA 24.376	Blood culture	France	Bordeaux	2021	0.5	0.06	4	NA	0.125	0.5	0.06	NA	0.03	0.03	This study
ERR15308136	CNRMA 24.605	Blood culture	France	Rennes	2024	0.25	0.125	2	NA	0.125	0.125	0.03	NA	0.015	0.015	This study
ERR15308137	CNRMA 24.757	Blood culture	France	Paris	2024	0.25	0.125	2	NA	0.06	0.125	0.125	NA	0.06	0.03	This study
ERR15308138	CNRMA 24.850	Blood culture	France	Bordeaux	2024	0.25	0.06	2	NA	0.125	0.25	0.06	NA	0.06	0.03	This study

WGS accession number	Strain number	Strain collection				MIC (mg/L)										Reference
		Sample type	Country	City	Year	AMB	FC	FLU	ITZ	VRC	POS	ISA	ANI	MICA	CAS	
ERR15308139	CNRMA 24.851	Blood culture	France	Bordeaux	2023	0.5	32	2	NA	0.125	0.06	0.03	NA	0.03	0.03	This study
ERR15308140	CNRMA 24.852	Blood culture	France	Bordeaux	2023	0.5	0.06	4	NA	0.125	0.5	0.06	NA	0.008	0.03	This study
ERR15308141	CNRMA 24.853	Blood culture	France	Bordeaux	2023	0.25	0.06	4	NA	0.125	0.25	0.25	NA	0.03	0.03	This study
ERR15308142	CNRMA 24.854	Blood culture	France	Bordeaux	2022	0.5	0.06	4	NA	0.125	0.5	0.06	NA	0.03	0.03	This study
ERR15308143	CNRMA 24.855	Blood culture	France	Bordeaux	2023	0.25	0.06	2	NA	0.125	0.25	0.06	NA	0.03	0.03	This study
ERR15308144	CNRMA 24.856	Skin	France	Bordeaux	2024	0.5	32	2	NA	0.125	0.125	0.03	NA	0.03	0.015	This study
ERR15308145	CNRMA 24.857	Cotton filter	France	Bordeaux	2024	0.25	0.06	4	NA	0.125	0.25	0.125	NA	0.06	0.03	This study
ERR15308146	CNRMA 24.858	Blood culture	France	Bordeaux	2024	0.5	0.06	2	NA	0.06	0.25	0.06	NA	0.03	0.015	This study
ERR15308147	CNRMA 24.917	Blood culture	France	Clermont-Ferrand	2024	0.5	0.125	4	NA	0.125	0.125	0.06	NA	0.03	0.03	This study
ERR15308148	CNRMA 24.970	Blood culture	France	Besancon	2024	0.25	0.125	4	NA	0.125	0.5	0.25	NA	0.03	0.008	This study
ERR15308149	CNRMA 5.433	Cardiac valv	France	Nice	2005	0.06	32	2	0.25	0.125	0.5	NA	NA	0.125	0.03	This study
ERR15308150	CNRMA 6.315	Blood culture	France	Paris	2006	0.06	0.125	2	0.125	0.125	0.25	NA	NA	0.06	0.03	This study
ERR15308151	CNRMA 7.1209	Blood culture	France	Paris	2007	0.06	0.125	4	0.06	0.06	0.25	NA	NA	0.06	0.03	This study
ERR15308152	CNRMA 8.315	Blood culture	France	Paris	2008	0.06	0.125	4	NA	0.25	1	NA	0.008	0.03	0.06	This study
ERR15308153	CNRMA 9.1011	Skin	France	Martinique	2009	0.06	0.125	4	NA	0.125	0.25	NA	0.008	0.03	0.06	This study
ERR15308154	CNRMA 9.540	Blood culture	France	Paris	2009	0.06	0.125	4	NA	0.125	0.5	NA	0.03	0.03	0.03	This study
ERR15308155	CNRMA 9.617	Blood culture	France	Paris	2008	0.125	0.125	2	NA	0.5	0.5	NA	0.015	0.03	0.06	This study

*AMB, amphotericin B; ANI, anidulafungin; CAS, caspofungin; FC, flucytosine; FLU, fluconazole; ISA, isavuconazole; ITZ, itraconazole; MIC, minimum inhibitory concentration; MICA, micafungine ; NA, not available; VRC, voriconazole; POS: posaconazole.

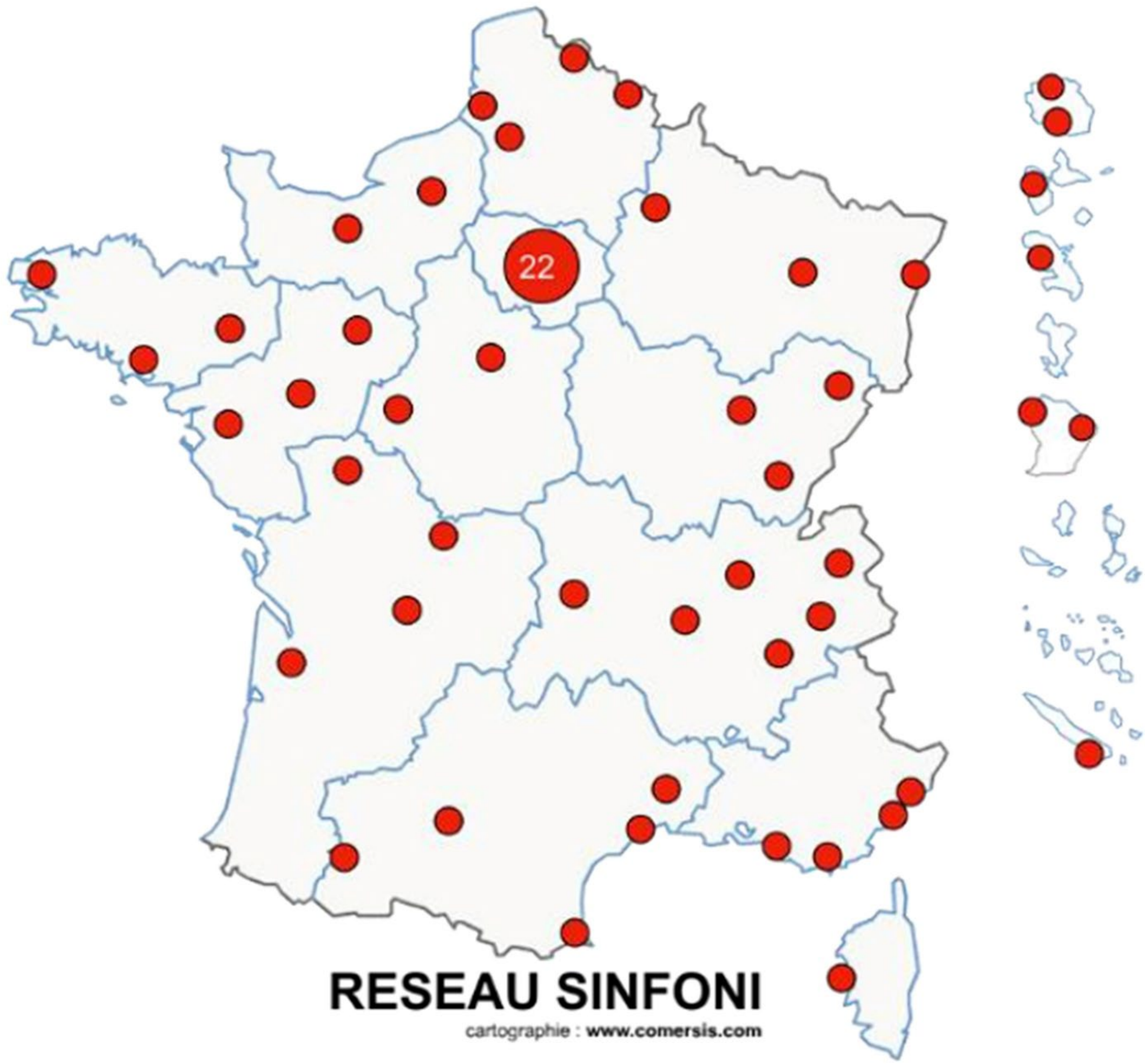

Appendix Figure 1. Map of French hospitals in mainland France and overseas territories participating in the RESSIF and SINFONI networks.

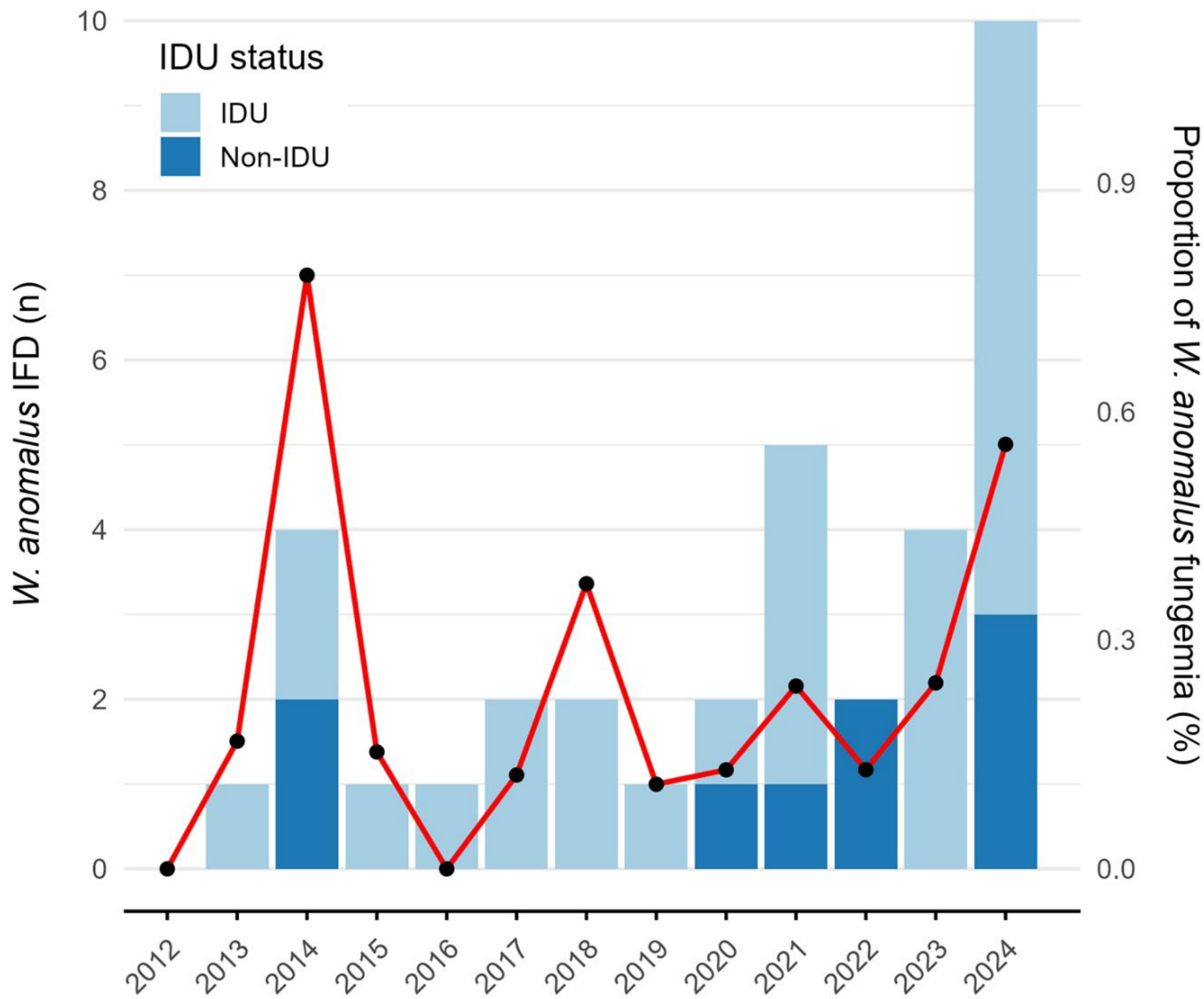

Appendix Figure 2. Annual incidence of *Wickerhamomyces anomalus* invasive infections and annual proportion of *W. anomalus* among all fungemias reported during the RESSIF and SINFONI national surveillance programs.

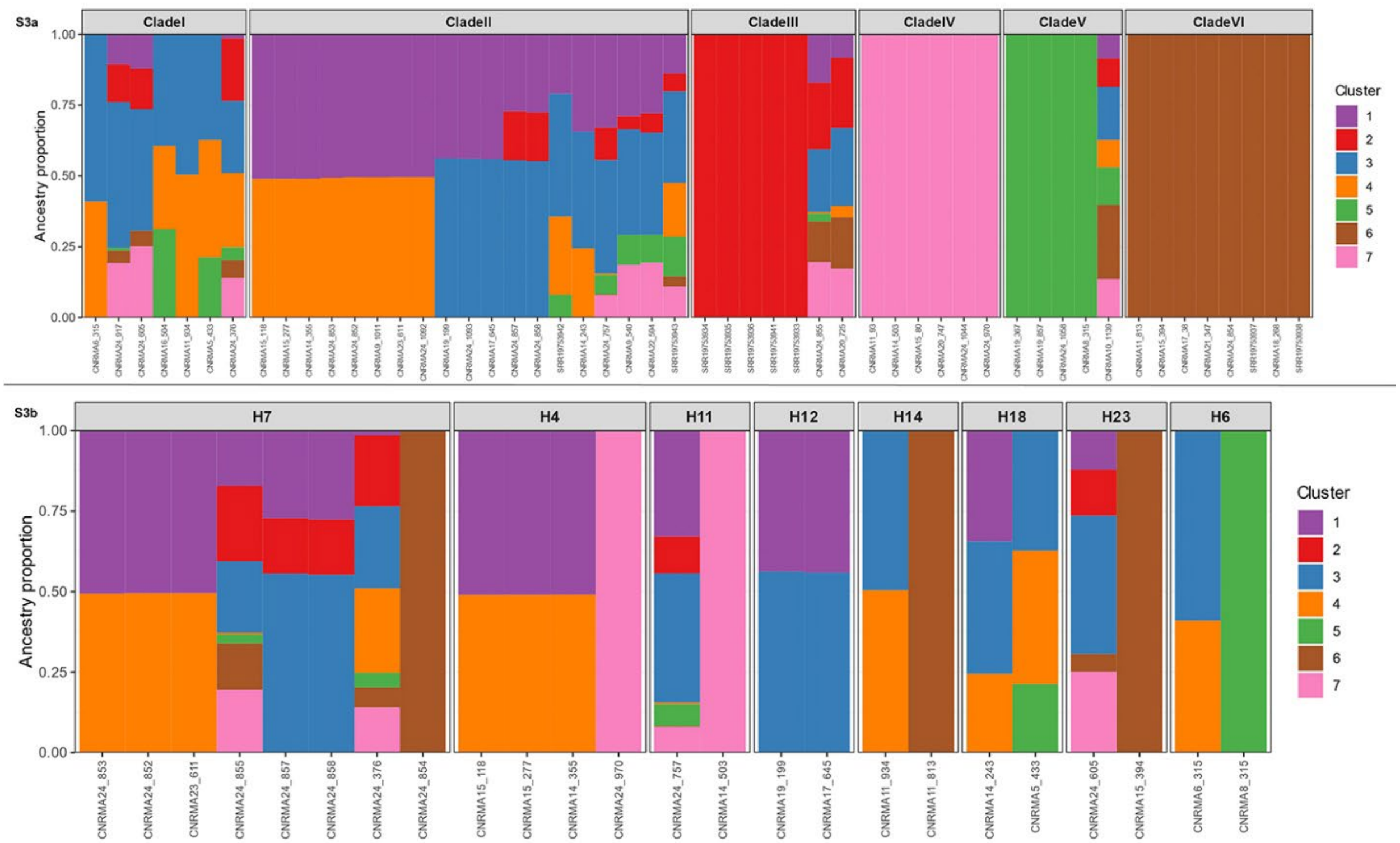

Appendix Figure 3. Population structure of *Wickerhamomyces anomalus* clinical isolates inferred by ADMIXTURE (K = 7), ordered by phylogenetic clade (S3a) or by center of isolation (S3b). Each vertical bar represents one isolate and is partitioned into colored segments proportional to the estimated ancestry contribution from each of the seven genetic clusters (K1–K7). The optimal number of clusters (K = 7) was determined by 10-fold cross-validation (minimum CV error). A) Isolates are grouped according to the six phylogenetic clades defined in the WGS tree. B) Only cities with at least two isolates are included. Isolates from the same city consistently belong to distinct genetic clusters, providing no evidence for city-level population structure.

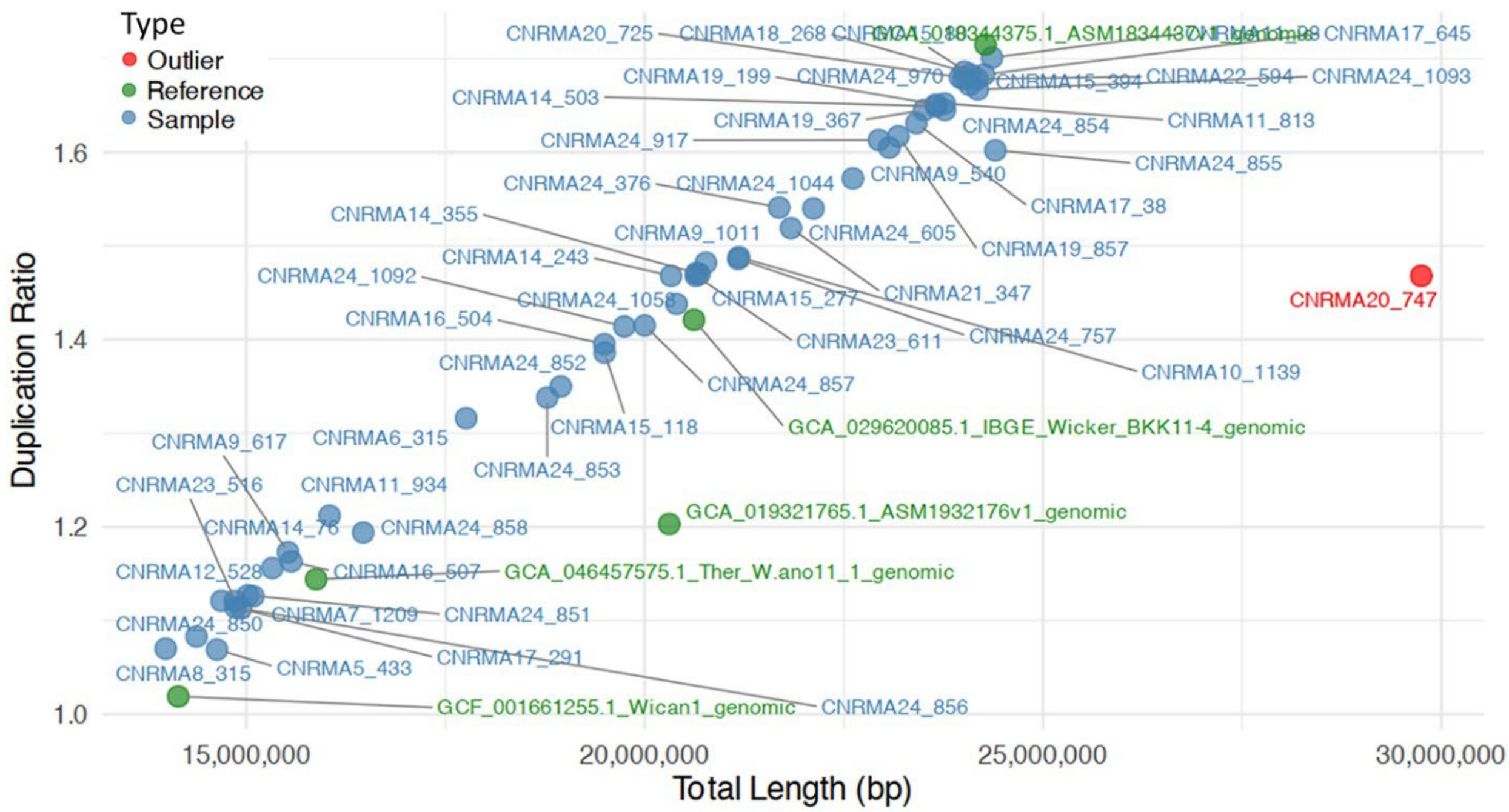

Appendix Figure 4. Duplication ratio plotted against assembly size for 53 *Wickerhamomyces. anomalus* de novo assemblies (blue and red) and publicly available genomes (green). Larger assemblies are associated with higher duplication ratios.

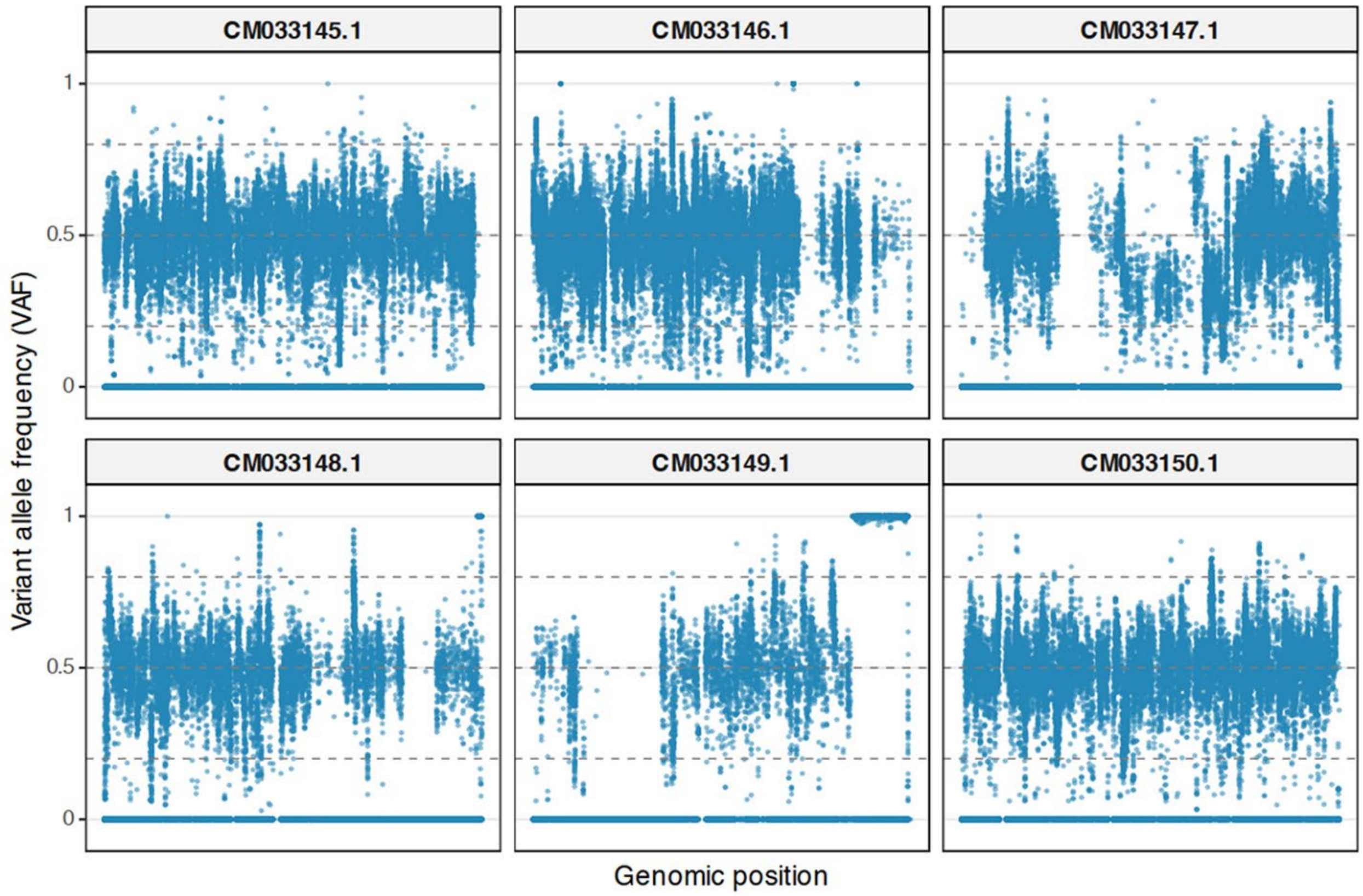

Appendix Figure 5. Variant allele frequency (VAF) for isolate CNRMA15.118.VAF represents the fraction of alternate allele reads over total coverage at each SNP. In diploid regions, variants cluster near 0.5 (heterozygous) or 0/1 (homozygous). Chromosome CM033147.1 shows an altered VAF pattern with heterozygous variants shifting to ≈0.33 and 0.67 in some regions, suggesting the presence of potential chromosomal alterations that warrant further investigation.
